# High throughput high content reverse genetics visual screens of ciliogenesis and cilia maintenance

**DOI:** 10.1186/2046-2530-1-S1-P49

**Published:** 2012-11-16

**Authors:** K Szymanska, G Wheway, S Natarajan, J Higgins, M Adams, DC Tomlinson, CA Johnson

**Affiliations:** 1 Leeds Institute of Molecular Medicine, UK

## 

Cilia are small, hair-like structures occurring on the apical surface of most of vertebrate cells. Defects in cilia cause a range of developmental phenotypes grouped into conditions called ciliopathies. Our aim is to dissect the structure and function of cilia and signalling pathways mediated by this organelle. To evaluate this, we are performing a high-throughput siRNA screen using siRNA pools (from the Dharmacon mouse genome siRNA library) targeting over 19,000 separate transcripts and immunofluorescence staining of ciliated mIMCD3 (transformed mouse inner medullary collecting duct) cells to determine cilia number, length and morphology. Secondary datasets from the screen will include measurements of cell size and morphology, nuclear morphology and cell cycle profiles. We have successfully set up a facility for high-throughput high-content imaging, optimized a reverse transfection protocol and validated a series of positive and negative controls. We are currently completing the analysis of candidate hits and expect to obtain several hundred positive hits from the whole screen. We will present the first dataset from this screen with a discussion of prioritization strategies for the validation of the most relevant and interesting candidate hits.

